# In silico identification and in vitro evaluation of MRPS30‐DT lncRNA and MRPS30 gene expression in breast cancer

**DOI:** 10.1002/cnr2.2114

**Published:** 2024-06-17

**Authors:** Nooshafarin Shirani, Roohallah Mahdi‐Esferizi, Reza Eshraghi Samani, Shahram Tahmasebian, Hajar Yaghoobi

**Affiliations:** ^1^ Clinical Biochemistry Research Center, Basic Health Sciences Institute Shahrekord University of Medical Sciences Shahrekord Iran; ^2^ Department of Medical Biotechnology School of Advanced Technologies, Shahrekord University of Medical Sciences Shahrekord Iran; ^3^ Department of General Surgery School of Medicine, Isfahan University of Medical Sciences Isfahan Iran

**Keywords:** bioinformatics analysis, breast cancer, MRPS30, MRPS30‐DT lncRNA

## Abstract

**Background:**

It has been reported that long non‐coding RNAs (lncRNAs) can play important roles in a variety of biological processes and cancer regulatory networks, including breast cancer.

**Aims:**

This study aimed to identify a novel upregulated lncRNA in breast cancer and its associated gene using bioinformatics analysis, and then evaluate their potential roles in breast cancer.

**Methods and Results:**

Extensive in silico studies were performed using various bioinformatics databases and tools to identify a potential upregulated breast cancer‐associated lncRNA and its co‐expressed gene, and to predict their potential roles, functions, and interactions. The expression level of MRPS30‐DT lncRNA and MRPS30 was assessed in both BC tissues and cell lines using qRT‐PCR technology. MRPS30‐DT lncRNA and MRPS30 were selected as target genes using bioinformatics analysis. We found that MRPS30‐DT and MRPS30 were significantly overexpressed in BC tissues compared with normal tissues. Also, MRPS30 showed upregulation in all three BC cell lines compared with HDF. On the other hand, MRPS30‐DT significantly increased in MDA‐MB‐231 compared with HDF. While the expression of MRPS30‐DT was significantly dropped in the resistance cell line MCF/MX compared to HDF and MCF7. Moreover, bioinformatics analysis suggested that MRPS30‐DT and MRPS30 may play a potential role in BC through their involvement in some cancer signaling pathways and processes, as well as through their interaction with TFs, genes, miRNAs, and proteins related to carcinogenesis.

**Conclusions:**

Overall, our findings showed the dysregulation of MRPS30‐DT lncRNA and MRPS30 may provide clues for exploring new therapeutic targets or molecular biomarkers in BC.

**Abbreviations**
ANLN: actin‐binding protein anillin
BC: breast cancer
BP: biological process
BRCAT54: breast cancer‐associated transcript 54
CC: cellular component
cDNA: complementary DNA
CENPF: centromere protein F
ceRNA: competing endogenous RNA network
DAP3: death associated protein 3
DEGs: differential expression genes
DILA1: cyclin D1‐interacting long noncoding RNA 1
DMEM/F12: Dulbecco's Modified Eagle Medium/Nutrient Mixture F‐12
EMT: epithelial‐Mesenchymal Transition
ER: estrogen receptor
FBS: fetal bovine serum
FDR: false discovery rate
FoxM1: forkhead box protein M1
FZD7: frizzled‐7
HDF: human dermal fibroblast cell line
HOTAIR: HOX transcript antisense RNA
HR: hormone receptor
KEGG: Kyoto encyclopedia of genes and genomes
LncRNAs: long non‐coding RNAs
MAGI2‐AS3: membrane associated guanylate kinase, WW and PDZ domain containing 2 (MAGI2) antisense RNA 3
MCM3AP‐AS1: minichromosome maintenance complex component 3 associated protein (MCM3AP) antisense RNA 1
MF: molecular function
MREs: MiRNA response elements
MRPL37: mitochondrial ribosomal protein L37
MRPS30: mitocondrial ribosomal protein S30
MRPS30‐DT: mitocondrial ribosomal protein S30‐Divergent transcript
NSCLC: non‐small cell lung cancer
PCA: principal component analysis
PDCD9: programmed cell death protein
Plks: polo‐like kinases
PPI: protein–protein interaction
qRT‐PCR: quantitative real‐time polymerase chain reaction
SD: standard deviation
STRING: search tool for the retrieval of interacting genes/proteins
TANRIC: the atlas of noncoding RNAs in cancer
TNBC: triple negative breast cancer
ZNF217: zinc Finger Protein 217

## 1 INTRODUCTION

According to the Global Cancer Observatory, breast cancer is a prevalent malignancy in women and is the leading cause of cancer‐related deaths among them.^1^, ^2^, ^3^ Despite advances in breast cancer diagnosis and treatment, the disease remains a challenge for global research.^4^ Therefore, it is necessary to identify novel biomarkers and molecular mechanisms in BC to enable early diagnosis and therapeutic strategies.^5^, ^6^

More than 98% of gene sequences consist of non‐coding genomes, so mutations in these regions of the genome are responsible for the majority of cancer phenotypes.^7^ lncRNAs are a class of non‐coding RNAs longer than 200 nucleotides, most of which are incapable of encoding proteins. lncRNAs are important factors in the regulation of pre‐transcriptional, transcriptional, and post‐transcriptional processes in biological functions and mechanisms. Numerous studies have demonstrated that lncRNAs play an important role in cancer both as oncogenes and as tumor suppressors by interacting with proteins, DNA, RNA, and other molecules. There is evidence that abnormal lncRNA expression influences the occurrence and progression of malignancy by affecting biological processes such as proliferation, metastasis, differentiation, apoptosis, invasion, angiogenesis, cell cycle, cell death, and miRNA silencing.^8^, ^9^, ^10^, ^11^, ^12^, ^13^ According to previous studies, an increasing number of lncRNAs are involved in the development of breast cancer. For example, high expression of the DILA1^*^ lncRNA was found to be associated with reduced degradation of the protein cyclin D1 (an important oncoprotein responsible for stimulating cancer cell proliferation) and poor prognosis in breast cancer patients undergoing tamoxifen therapy.^14^ Moreover, LncRNA MCM3AP‐AS1 was reported to be upregulated in breast cancer and higher expression was associated with cell proliferation, migration, invasion, and tumor growth through modulation of the MCM3AP‐AS1/miR‐28‐5p/CENPF pathway.^15^ A study revealed that high expression of HOTAIR lncRNA in breast cancer cells and tissues induced cell proliferation, migration, invasion and EMT in breast cancer cells via sponging miR‐129‐5p to elevate the expression of FZD7.^16^ According to an investigation, the downregulation of MAGI2‐AS3 lncRNA in breast cancer showed the tumor suppressive role of MAGI2‐AS3 by affecting Fas and FasL signaling pathways.^17^ However, the importance of most lncRNAs and their underlying biological mechanisms in the control of breast cancer remain largely unexplored.

In the present study, we aimed to identify the most upregulated lncRNAs involved in breast cancer and select one of them and its associated gene through various bioinformatics analyses performed using databases and bioinformatics websites. Hence, we selected MRPS30‐DT as a novel lncRNA with limited information about its role in breast cancer and MRPS30 as its co‐expressed gene. We then conducted further bioinformatics analyses to predict the possible role and mechanism of MRPS30‐DT and MRPS30 in BC. The lncRNA MRPS30‐DT or BRCAT54 is an antisense lncRNA, located on chromosome 5p12.

The lncRNA MRPS30‐DT or BRCAT54 is an antisense lncRNA, located on chromosome 5p12.^18^ Based on a previous study, the results of microarray analysis of three pairs of tumor and normal breast tissues showed upregulation of lncRNA MRPS30‐DT in the tumor tissue. In addition, MRPS30‐DT and Jab1 were overexpressed in breast cancer samples as detected by in situ hybridization and immunohistochemistry, respectively, compared to normal tissue. Furthermore, the knockdown of MRPS30‐DT was found to suppress cell proliferation, migration, and invasion in MCF‐7 and MDA‐MB‐231 breast cancer cells while inducing apoptosis. The expression of Jab1 in breast cancer cell lines was also significantly reduced by knocking down MRPS30‐DT.^19^ Moreover, the expression levels of this lncRNA were found to be significantly increased in NSCLC, and it could inhibit tumorigenesis of NSCLC.^20^

Mitochondrial ribosomal proteins are not only involved in protein biosynthesis, but also play a crucial role in the cell cycle, apoptosis, and tumorigenesis.^21^, ^22^, ^23^ MRPS30 is one of more than 70 protein components of mitochondrial ribosomes that are encoded by the nuclear genome and contribute to protein synthesis in mitochondria. It is located on chromosome 5p12‐q11 and is also called PDCD9.^23^ Previous studies have indicated that the MRPS30 gene disrupts cell behavior in breast cancer cells, contributing to the development of cancer.^24^ In a genome‐wide association study on breast cancer predisposition, two SNPs were identified on 5p12, with MRPS30 being the closest gene.^25^ Furthermore, according to a review study, MRPS30 was a genomic region associated with breast cancer risk.^26^ In addition, the 5p12 variant rs10941679 has been shown to increase the risk of developing estrogen receptor‐positive breast cancer through the regulation of FGF10 and MRPS30.^27^

Following the in silico approach, we also examined the expression of MRPS30‐DT lncRNA and the MRPS30 gene in BC tissues and BC cell lines (MCF7, MCF7/MX, MDA‐MB‐231) compared with adjacent non‐tumor tissues and the HDF^†^ cell line with the aim of investigating the possible role of these genes in breast cancer. The results of the present study may contribute to the discovery of new and effective therapeutic targets to combat BC. Moreover, our study provides a background on some molecular mechanisms involved in breast cancer based on a comprehensive bioinformatics analyses.

## 2 MATERIALS AND METHODS

### 2.1 Bioinformatics analyses

In order to select the genes of the current study and their roles in BC, several web servers and databases were used (Table 1). First, 12 727 gene expression of long noncoding RNA (lncRNA) from 837 breast cancer samples and 105 normal breast samples were downloaded from TANRIC database. The expression data were then imported into the iDEP website, and analyzes such as heatmap, PCA, and DEGs were subsequently performed. First, we determined lncRNAs with lgFC >1 as upregulated lncRNAs and those with lgFC < −1 as downregulated lncRNAs and considered the false discovery rate (FDR) < 0.01 in both cases. Scatter plot, MA plot, and volcano plot were implemented by the iDEP website on the differentially expressed gene analysis. In the next step, we collected lncRNAs involved in breast cancer using the LncBook database. Finally, we shared the two lists of lncRNAs (upregulated lncRNAs according to DEG analysis and lncRNAs involved in breast cancer based on LncBook) with the VENNY 2.1.0 diagram.

**TABLE 1:** Bioinformatics databases and tools used in this study.

Databases and websites	URL	Reference
TANRIC	https://www.tanric.org	28
iDEP.93	http://bioinformatics.sdstate.edu/idep93/	29
LncBook	https://bigd.big.ac.cn/lncbook	30
VENNY 2.1.0	https://bioinfogp.cnb.csic.es/tools/venny	31
lncHUB	https://maayanlab.cloud/lnchub	32
Appyters	https://appyters.maayanlab.cloud/Enrichment_Analysis_Visualizer	33
GeneMANIA	http://genemania.org	34
STRING	https://string‐db.org	35, 36
LncBase v3	https://diana.e‐ce.uth.gr/lncbasev3/home	37
TarBase v8	https://dianalab.e‐ce.uth.gr/html/diana/web/index.php?r=tarbasev8	38

After selecting the candidate lncRNA, we investigated its association with some clinicopathological characteristics of the patients (stage, ER status, and PR status) using the TANRIC online website.

The lncHUB website was used to predict the gene with the highest score that is associated with the candidate lncRNA. Then the 50 top co‐expressed genes extracted from lncHUB, were used as input for computing different enrichment analyses with the Appyters website. Finally, The GeneMANIA and STRING^‡^ websites were used to anticipate the physical and protein interactions of the selected associated gene with the highest ranking. In addition, we evaluated the correlation between the selected lncRNA and its most associated gene by TANRIC. We also downloaded the possible target miRNAs of MRPS30‐DT lncRNA and MRPS30 identified in Homo sapiens using LncBase and TarBase databases, respectively, and then shared the miRNAs derived from these two databases using VENNY diagram to find common miRNAs between them.

### 2.2 Patient and clinical tissue collection

We collected breast cancer tissue and adjacent normal tissue from 41 patients who underwent breast cancer surgery at Kashani Hospital in Chaharmahal and Bakhtiari Province and Omid Hospital in Isfahan Province between February 2020 and March 2021. The clinicopathologic features of these patients were recorded simultaneously, and all specimens were also evaluated by a pathologist to ensure diagnosis. Each patient signed an informed consent form. Patients diagnosed with breast cancer by clinical tests and approved by the surgeon and oncologist were included; those who did not meet these criteria were excluded. Samples were immediately placed in cryogenic tubes containing RNA later solution (Yekta Tajhiz Azma) and stored in a freezer at −80°C until processed. The study was approved by the Ethics Committee of the Shahrekord University of Medical Sciences (Code: IR.SKUMS.REC.1398.209).

### 2.3 Cell culture

Three human breast cancer cell lines (MCF‐7, MDA‐MB‐231, and MCF‐7/MX), and a human epidermal fibroblast (HDF) cell line as a normal cell line were provided by National Cell Bank of Iran (Pasteur Institute, Iran), and then harvested in DMEM/F12 medium containing 5% (FBS), 1% penicillin and 1% streptomycin at 37°C in a humidified atmosphere with 5% CO₂. Additionally, cultured cells were tested for Mycoplasma infections.

### 2.4 RNA extraction and quantitative real‐time PCR (qRT‐PCR)

Total RNA was extracted from tissues and cell lines using RNX‐Plus solution (a guanidine/phenol solution), and Hybrid‐R kit (GeneAll, Seoul, South Korea) according to the manufacturer's protocol. The quantity and quality of the extracted RNA was determined using the NanoDrop™ 2000 spectrophotometer and gel electrophoresis. Subsequently, the cDNA was synthesized using the ExcelRT™ Reverse Transcription Kit (SMOBio, Taiwan) according to the manufacturer's instructions. The relative expression of MRPS30‐DT lncRNA and the associated MRPS30 gene was detected in all samples and cell lines by qRT‐PCR SYBR Green (Yekta Tajhiz Azma). Quantitative real‐time PCR was conducted in duplicate in the Rotor Gene 3000 Corbett Real‐Time PCR System. The β‐actin gene was also used as a normalizer, and the fold‐change of gene expression was determined using the 2^−ΔΔCt^ method. Priming design was performed under optimal primer conditions by Clustal Omega (https://www.ebi.ac.uk/Tools/msa/clustalo), Primer Blast (https://www.ncbi.nlm.nih.gov/tools/primer-blast), and Oligoanalyzer (https://www.idtdna.com/pages/tools/oligoanalyzer) websites. The primers were listed in Table 2:

**TABLE 2:** The nucleotide sequences of primers used in the current study.

Primers	Primer sequence	Primer length	PCR product
MRPS30	Forward: 5′ AGGGAAAGGCTTTTGAGAC 3′ Reverse: 5′ ATCTGCTTCACTCCAGAATC 3′	19 20	129 bp
MRPS30‐DT	Forward: 5′ TACACATTTCCCACCTTGC 3′ Reverse: 5′ CACCTCCAAACACCATCAC 3′	19 19	108 bp
β‐actin	Forward: 5′‐TCATGAAGTGTGACGTGGAC‐3′ Reverse: 5′‐CAGGAGGAGCAATGATCTTG‐3′	20 20	156 bp

### 2.5 Statistical analysis

All data are presented as the mean ± SD. Graph Pad Prism v.9.2.0 software was used for photo drafting and statistical analysis. A two‐tailed t‐test was chosen for comparisons of continuous variables between two groups, and a one‐way analysis (ANOVA) was used when three or more groups were compared. Pearson correlation test was also performed to investigate the correlation between parameters. *p* < .05 was considered statistically significant.

## 3 RESULTS

### 3.1 Bioinformatics analyses

Analyses like Heatmap, PCA, and DEG were conducted on the data obtained from the TANRIC database using the iDEP website. At first, to ensure the validity of the data used in TANRIC, we performed Heatmap and PCA analyses (Figure S1). According to Heatmap and PCA, it was found that the expression profiles of different lncRNAs were able to distinguish tumor samples from normal tissue samples and both demonstrated the high quality of the data used.

### 3.2 DEG analysis

**FIGURE 1:**
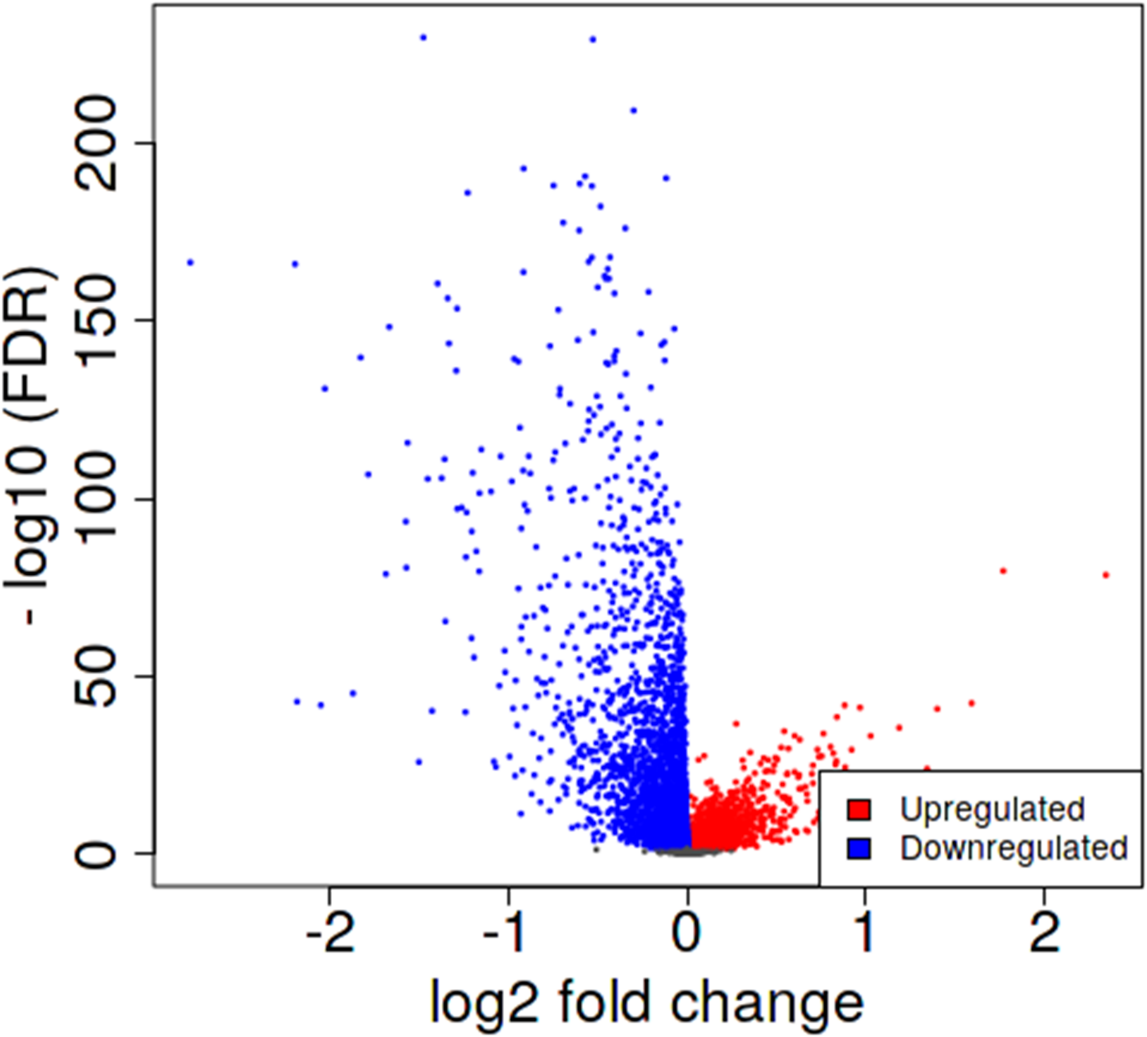
Volcano plot. This plot shows the differential expression of lncRNAs between tumor and normal tissues. In this plot, the log2 fold change (cut‐off = ±1) was plotted against the ‒log10 FDR (cut‐off = 2). Red and blue dots represent upregulated and downregulated lncRNAs, respectively, and gray dots represent other lncRNAs without significant change.

Through DEG analysis of the iDEP website, we found a total of 64 lncRNAs that exhibited differential expression in tumor tissues compared with normal tissues, including 18 upregulated LncRNAs and 46 downregulated LncRNAs. The cut‐off point for differential expression was lgFC >1 or lgFC < −1, where the FDR was <0.01, as shown in the volcano plot (Figure 1). Table S1 lists the dysregulated lncRNAs in tumor tissues compared to normal tissues. The scatter plot and the MA plot are shown in Figure S2. Taken together, these results suggested that these dysregulated lncRNAs may have specific functions in breast cancer development.

### 3.3 Identification of MRPS30‐DT lncRNA and MRPS30

We identified 129 lncRNA involved in breast cancer through the lncBook database. After sharing upregulated lncRNAs from the TANRIC database based on DEG analysis and breast cancer‐related lncRNAs from the lncBook, we found two lncRNAs named MRPS30‐DT and DSCAM‐AS1 (Figure S3).

Then, MRPS30‐DT was selected as a candidate lncRNA that limited experiments had been conducted on its role in breast cancer. Consequently, we listed the most co‐expressed genes predicted to be associated with MRPS30‐DT using the lncHUB database, and we chose the MRPS30 gene as the most related gene. The 50 most frequently co‐expressed genes with MRPS30‐DT are listed in Table S2.

### 3.4 Association of MRPS30‐DT lncRNA with clinicopathological features via TANRIC

**FIGURE 2:**
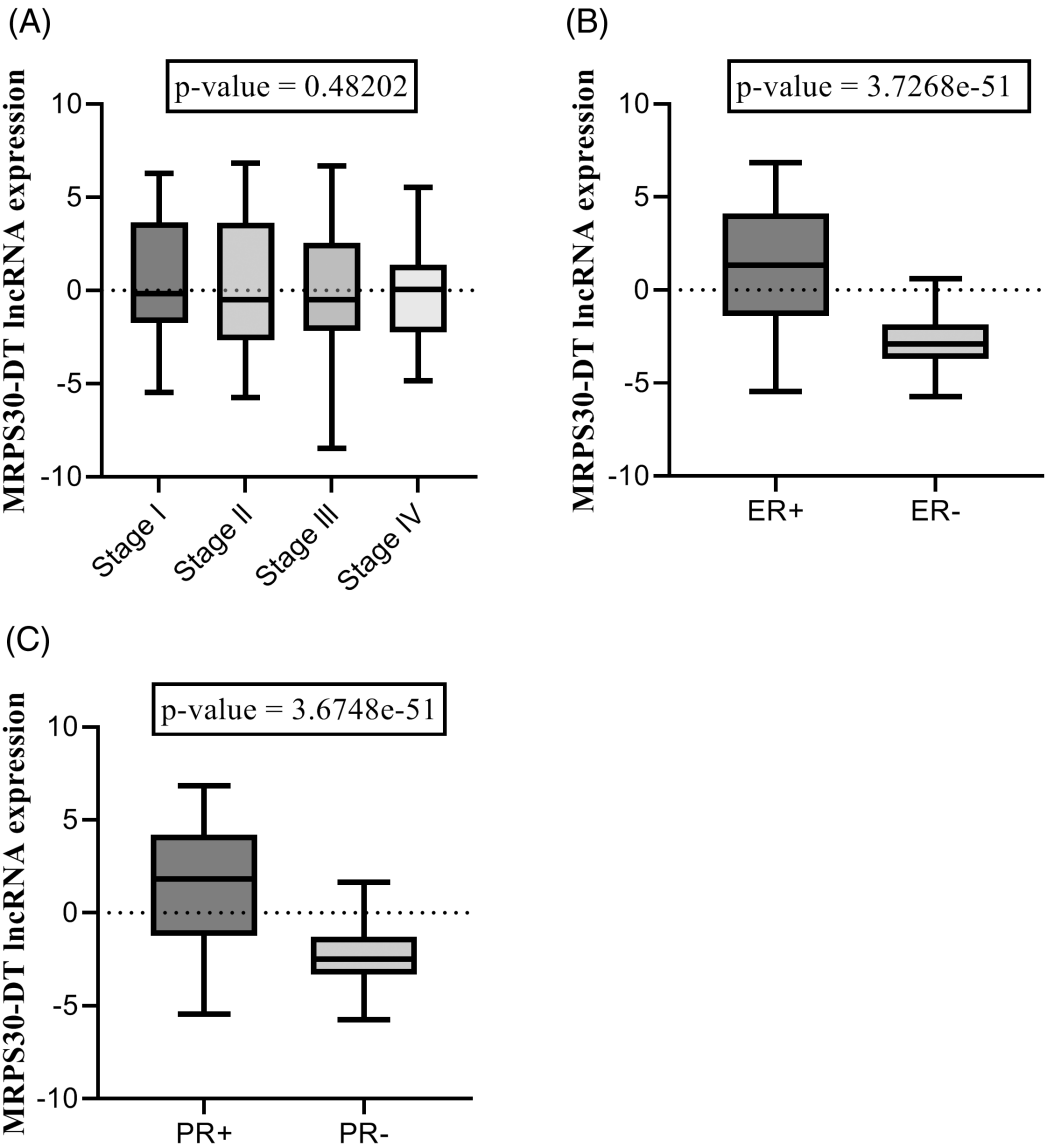
MRPS30‐DT lncRNA association with clinicopathological characteristics via TANRIC subtype analyses. (A) Association with tumor stage (*p*‐value = 0.48202). (B) Association with ER status (ER+ [median]: 1.3374; ER− [median]: −2.9101, *p*‐value = 3.7268e−51). (C) Association with PR status (PR+ [median]: 1.8224; PR− [median]: −2.487, *p*‐value = 3.6748e−51).

The association of MRPS30‐DT lncRNA with some clinicopathological features, including stage, ER status, and PR status, is shown in Figure 2. The results revealed no significant association between the expression of MRPS30‐DT and tumor stage (Figure 2A, *p*‐value = .48202), whereas there was a significant direct association between the expression of MRPS30‐DT and the presence of estrogen and progesterone receptors. The expression of MRPS30‐DT was higher in the ER + (Figure 2B, *p‐*value = 3.7268e‐51) and PR + (Figure 2C, *p*‐value = 3.6748e‐51) groups than in the ER− and PR− groups.

### 3.5 Enrichment analyses

To investigate the pathways, functions, and diseases associated with MRPS30‐DT lncRNA, an enrichment analysis of the top 50 genes co‐expressed with this lncRNA by pathway, transcription factor, disease, and cell type was performed on the appyters website. KEGG pathway enrichment analysis (KEGG 2021) revealed that the co‐expressed genes were enriched in six pathways. The most enriched pathways included the Hedgehog pathway and the prolactin pathway (Table 3).

**TABLE 3:** Different enrichment analyses of co‐expressed genes with MRPS30‐DT lncRNA.

Enrichment analysis	Term	*p‐value*	q‐value	Overlap genes
**KEGG 2021 human**	Hedgehog signaling pathway	.000371	0.025986	SCUBE2, SPOPL, BTRC
	Prolactin signaling pathway	.013274	0.352112	PRLR, ESR1
	Th17 cell differentiation	.029403	0.352112	GATA3, IL6ST
	Cytokine‐cytokine receptor interaction	.037434	0.352112	IL6ST, BMPR1B, PRLR
	Pathways in cancer	.043347	0.352112	AGTR1, IL6ST, ESR1, FGF10
	Signaling pathways regulating pluripotency of stem cells	.049751	0.352112	IL6ST, BMPR1B
**GO biological process 2021**	Neuron differentiation (GO:0030182)	.000073	0.053047	FOXA1, UGCG, XBP1, IRX5, LMX1B
	Response to interleukin‐4 (GO:0070670)	.000272	0.053047	XBP1, GATA3
	Cellular response to interleukin‐4 (GO:0071353)	.000272	0.053047	XBP1, GATA3
	Mesonephric development (GO:0001823)	.000332	0.053047	GATA3, FGF10
	Kidney development (GO:0001822)	.000715	0.080284	AGTR1, GATA3, FGF10
	Positive regulation of T cell activation (GO:0050870)	.000875	0.080284	XBP1, GATA3, IL6ST
	Regulation of cartilage development (GO:0061035)	.000913	0.080284	TRPS1, BMPR1B
	Positive regulation of lymphocyte differentiation (GO:0045621)	.001131	0.080284	XBP1, GATA3
	Dopaminergic neuron differentiation (GO:0071542)	.001131	0.080284	FOXA1, LMX1B
	Dorsal/ventral pattern formation (GO:0009953)	.001632	0.094812	LMX1B, BMPR1B
**GO molecular function 2021**	Oncostatin‐M receptor activity (GO:0004924)	.000091	0.004062	IL6ST, PRLR
	Leukemia inhibitory factor receptor activity (GO:0004923)	.000091	0.004062	IL6ST, PRLR
	Ciliary neurotrophic factor receptor activity (GO:0004897)	.000170	0.004852	IL6ST, PRLR
	Ciliary neurotrophic factor receptor binding (GO:0005127)	.000218	0.004852	IL6ST, PRLR
	Double‐stranded DNA binding (GO:0003690)	.001109	0.018340	FOXA1, ZNF396, XBP1, IRX5, LMX1B, GATA3, ESR1
	Cytokine receptor activity (GO:0004896)	.001389	0.018340	GFRA1, IL6ST, PRLR
	RNA polymerase II transcription regulatory region sequence‐specific DNA binding (GO:0000977)	.001710	0.018340	FOXA1, ZNF396, XBP1, ZNF92, IRX5, TRPS1, LMX1B, GATA3, ESR1, ZNF552
	Sequence‐specific DNA binding (GO:0043565)	.001782	0.018340	FOXA1, ZNF396, XBP1, IRX5, LMX1B, GATA3, ESR1
	Sequence‐specific double‐stranded DNA binding (GO:1990837)	.001855	0.018340	FOXA1, ZNF396, XBP1, IRX5, LMX1B, GATA3, ESR1
	Cis‐regulatory region sequence‐specific DNA binding (GO:0000987)	.007222	0.058435	FOXA1, ZNF396, XBP1, ZNF92, IRX5, GATA3, ESR1, ZNF552
**GO cellular component 2021**	Protein kinase complex (GO:1902911)	.017372	0.28222	NEK10
	HFE‐transferrin receptor complex (GO:1990712)	.019829	0.28222	BMPR1B
	Transferase complex, transferring phosphorus‐containing groups (GO:0061695)	.022281	0.28222	NEK10
**ChEA 2016**	ZNF217 24 962 896 ChIP‐Seq MCF‐7 Human	9.765695e−18	5.644571e−15	FOXA1, CLSTN2, GATA3, AFF3, TMEM26, UGCG, TRPS1, STC2, SLC39A6, AGR3, RGS22, NBPF4, XBP1, ELOVL5, TPRG1, TBC1D9, GFRA1, KCTD3, PRLR, ESR1, PKIB, SPOPL, LMX1B, IL6ST, FSIP1, BMPR1B, FGF10
	FOXM1 26 456 572 ChIP‐Seq MCF‐7 Human	6.572979e−11	1.899591e−08	FOXA1, XBP1, CLSTN2, ELOVL5, TPRG1, TBC1D9, KCTD3, MRPS30, AFF3, PRLR, ESR1, UGCG, PKIB, TRPS1, AGTR1, SLC39A6, NEK10, SPOPL, AGR3, RGS22, LMX1B, IL6ST, FGF10
	ARNT 22903824 ChIP‐Seq MCF‐7 Human	6.374735e−06	1.228199e−03	CT62, CLSTN2, TPRG1, CCDC74A, TRPS1, STC2, AGR3, COX6C, FSIP1, BMPR1B, PRLR, ESR1
	GATA3 24 758 297 ChIP‐Seq MCF‐7 Human	1.694909e−05	1.981995e−03	IRX5, ELOVL5, TPRG1, ELP2, GFRA1, KCTD3, COX6C, ESR1, UGCG, CT62, RABEP1, AGR3, RGS22, FSIP1, BTRC, ZNF552
	SUZ12 18 692 474 ChIP‐Seq MEFs Mouse	1.714528e−05	1.981995e−03	PTPRT, SCUBE2, FOXA1, PKIB, IRX5, CLSTN2, STC2, SUSD3, TBC1D9, GFRA1, LMX1B, GATA3
	AR 22383394 ChIP‐Seq PROSTATE CANCER Human	3.034267e−05	2.923010e−03	KCNE4, TPRG1, GATA3, AFF3, PRLR, KIAA0040, TMEM26, CCDC74A, SLC39A6, NEK10, AGR3, RGS22, LMX1B, FSIP1, BMPR1B
	AHR 22903824 ChIP‐Seq MCF‐7 Human	4.635909e−05	3.827936e−03	CT62, TPRG1, CCDC74A, TRPS1, STC2, AGR3, COX6C, FSIP1, ESR1
	RUNX 20019798 ChIP‐Seq JUKART Human	1.326009e−04	9.580416e−03	XBP1, ELOVL5, TPRG1, LMX1B, GATA3, AFF3, BTRC, KIAA0040
	TFAP2C 20 629 094 ChIP‐Seq MCF‐7 Human	1.515335e−04	9.731820e−03	FOXA1, PKIB, CT62, RABEP1, ELOVL5, TPRG1, TBC1D9, KCTD3, LMX1B, PRLR, ESR1
	SUZ12 18 974 828 ChIP‐Seq MESCs Mouse	1.963825e−04	1.135091e−02	PTPRT, FOXA1, IRX5, CLSTN2, TBC1D9, GFRA1, GATA3, AFF3, SCUBE2, PKIB, STC2, SUSD3, LMX1B, FGF10
**DisGeNET**	Mammary neoplasms, experimental	.000003	0.003282	TRPS1, AGTR1, GATA3, IL6ST, PRLR, ESR1
	Renal cell dysplasia	.000004	0.003282	GATA3, LMX1B, ESR1
	Breast carcinoma	.000009	0.003695	FOXA1, MRPS30, GATA3, AFF3, SCUBE2, UGCG, TRPS1, STC2, SLC39A6, SUSD3, AGR3, BTRC, XBP1, IRX5, TBC1D9, GFRA1, PRLR, ESR1, PKIB, AGTR1, NEK10, MAGED2, LMX1B, IL6ST, FSIP1, BMPR1B, FGF10
	Mammary neoplasms	.000009	0.003695	FOXA1, XBP1, TBC1D9, MRPS30, GATA3, AFF3, PRLR, ESR1, SCUBE2, UGCG, RABEP1, TRPS1, STC2, AGTR1, SLC39A6, SUSD3, AGR3, FGF10
	Carcinoma	.000016	0.005029	FOXA1, TRPS1, GATA3, IL6ST, PRLR, ESR1
	Mammary carcinoma, animal	.000030	0.006913	TRPS1, GATA3, IL6ST, PRLR, ESR1
	Animal mammary neoplasms	.000030	0.006913	TRPS1, GATA3, IL6ST, PRLR, ESR1
	Breast adenocarcinoma	.000061	0.012214	FOXA1, UGCG, TBC1D9, GATA3, ESR1
	Carcinomatosis	.000077	0.013534	TRPS1, GATA3, IL6ST, PRLR, ESR1
	Carcinoma, spindle‐cell	.000105	0.016624	TRPS1, GATA3, IL6ST, PRLR, ESR1

*Note*: The top ten of each enrichment analysis with a *p*‐value <.05 are shown (The q‐value is an adjusted *p*‐value calculated using the Benjamini‐Hochberg method to correct multiple hypothesis testing).

GO term enrichment analysis (GO 2021) included the categories of biological process (BP), molecular function (MF), and cellular component (CC). As shown in File S1, a total of 243 GO terms of the biological process are listed. According to Table 3, the genes co‐expressed with MRPS30‐DT played a significant role in biological processes such as neuron differentiation and response to interleukin‐4. The molecular function is represented in File S2 by 36 GO terms. Furthermore, oncostatin‐M receptor activity and leukemia inhibitory factor receptor activity contributed significantly to the molecular function of these genes (Table 3). According to the CC terms, co‐expressed genes were enriched in three cellular components, including protein kinase complex, HFE‐transferrin receptor complex, and transferase complex (Table 3).

Transcriptional enrichment analysis (ChEA 2016) of genes co‐expressed with MRPS30‐DT revealed 77 enriched transcription factors (File S3). Table 3 shows that genes co‐expressed with MRPS30‐DT were notably associated with transcription factors such as ZNF217 and FoxM1.

According to the disease enrichment analysis (DisGeNET) in File S4, genes co‐expressed with MRPS30‐DT were associated with 480 diseases. The most enriched diseases included mammary neoplasms, and renal cell dysplasia, and in general, the co‐expressed genes were related to cancer, especially breast cancer (Table 3). For all enrichment analyses, the *p*‐value was considered to be less than .05.

### 3.6 Gene–gene and PPI ^§^ network of the MRPS30 gene

The database STRING showed some interactions between MRPS30 and some proteins that interfere in apoptosis, such as DAP3 and MRPL37 (Figure S4a). Based on the GeneMANIA website interaction network, MRPS30 interacted most strongly with MLPL37 and MRPL28, respectively (Figure S4b).

### 3.7 Correlation between expression levels of MRPS30‐DT lncRNA and MRPS30 using TANRIC

**FIGURE 3:**
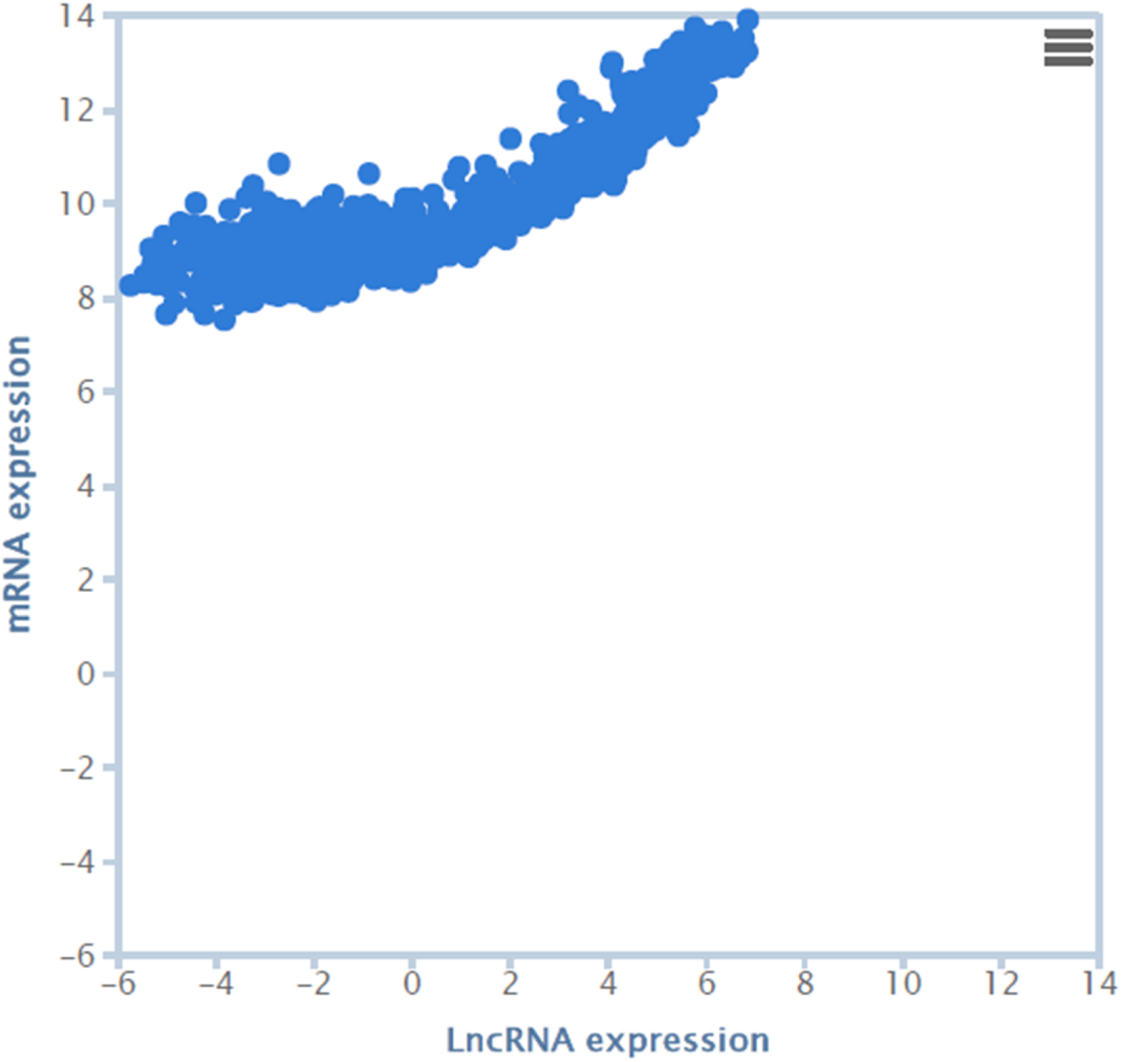
Correlation between expression levels of MRPS30‐DT lncRNA and MRPS30 mRNA using TANRIC mRNA correlation analysis.

Correlation analysis in the TANRIC database showed a significant positive correlation between the expression levels of MRPS30‐DT lncRNA and the MRPS30 gene (*p*‐value = 6.75e‐221; *r* = .842; Figure 3).

### 3.8 Prediction of common possible miRNAs between MRPS30‐DT lncRNA and MRPS30

The total list of MRPS30‐DT lncRNA‐miRNA interactions contained 42 miRNAs with high confidence levels retrieved from the LncBase database, and we also obtained the 25 MRPS30 mRNA‐miRNA interactions from the TarBase database. Finally, we found 5 miRNAs in common between MRPS30‐DT lncRNA and MRPS30 (Table 4).

**TABLE 4:** Common possible miRNAs between MRPS30‐DT lncRNA and MRPS30 using LncBase and TarBase databases.

Rank	miRNAs‐MRPS30‐DT lncRNA interaction from LncBase database	Rank	miRNAs‐ MRPS30 mRNA interaction from TarBase database	Rank	Common miRNAs between two databases
1	hsa‐let‐7a‐5p	1	hsa‐miR‐33a‐5p	1	hsa‐let‐7b‐5p
2	hsa‐let‐7b‐5p	2	hsa‐miR‐33b‐5p	2	hsa‐miR‐1‐3p
3	hsa‐let‐7c‐5p	3	hsa‐miR‐148a‐3p	3	hsa‐miR‐16‐5p
4	hsa‐let‐7d‐5p	4	hsa‐miR‐148b‐3p	4	hsa‐miR‐34a‐5p
5	hsa‐let‐7e‐5p	5	hsa‐miR‐126‐5p	5	hsa‐miR‐7‐5p
6	hsa‐let‐7f‐5p	6	hsa‐miR‐152‐3p		
7	hsa‐let‐7g‐5p	7	hsa‐miR‐3605‐5p		
8	hsa‐let‐7i‐5p	8	hsa‐miR‐1246		
9	hsa‐miR‐1‐3p	9	hsa‐miR‐150‐3p		
10	hsa‐miR‐103a‐3p	10	hsa‐miR‐183‐3p		
11	hsa‐miR‐107	11	hsa‐miR‐200c‐3p		
12	hsa‐miR‐128‐3p	12	hsa‐miR‐3934‐5p		
13	hsa‐miR‐1301‐3p	13	hsa‐miR‐548L		
14	hsa‐miR‐130a‐5p	14	hsa‐miR‐7‐5p		
15	hsa‐miR‐139‐5p	15	hsa‐miR‐1‐3p		
16	hsa‐miR‐15a‐5p	16	hsa‐miR‐34a‐5p		
17	hsa‐miR‐15b‐5p	17	hsa‐let‐7b‐5p		
18	hsa‐miR‐16‐5p	18	hsa‐miR‐101‐3p		
19	hsa‐miR‐17‐5p	19	hsa‐miR‐10b‐5p		
20	hsa‐miR‐181a‐5p	20	hsa‐miR‐124‐3p		
21	hsa‐miR‐183‐5p	21	hsa‐miR‐16‐5p		
22	hsa‐miR‐19a‐3p	22	hsa‐miR‐182‐5p		
23	hsa‐miR‐19b‐3p	23	hsa‐miR‐34a‐5p		
24	hsa‐miR‐20b‐5p	24	hsa‐miR‐33b‐5p		
25	hsa‐miR‐210‐3p	25	hsa‐miR‐33a‐5p		
26	hsa‐miR‐218‐5p				
27	hsa‐miR‐221‐3p				
28	hsa‐miR‐222‐3p				
29	hsa‐miR‐23a‐3p				
30	hsa‐miR‐27a‐3p				
31	hsa‐miR‐27b‐3p				
34	hsa‐miR‐29b‐3p				
35	hsa‐miR‐29c‐3p				
36	hsa‐miR‐30c‐5p				
37	hsa‐miR‐320a‐3p				
38	hsa‐miR‐34a‐5p				
39	hsa‐miR‐423‐5p				
40	hsa‐miR‐7‐5p				
41	hsa‐miR‐93‐5p				
42	hsa‐miR‐98‐5p				

### 3.9 Expression assays

In an effort to experimentally assess the expression patterns of MRPS30‐DT lncRNA and the MRPS30 gene, we used qRT‐PCR technology.

### 3.10 Expression levels of MRPS30‐DT lncRNA and MRPS30 in breast cancer tumor tissue and adjacent normal tissues

**FIGURE 4:**
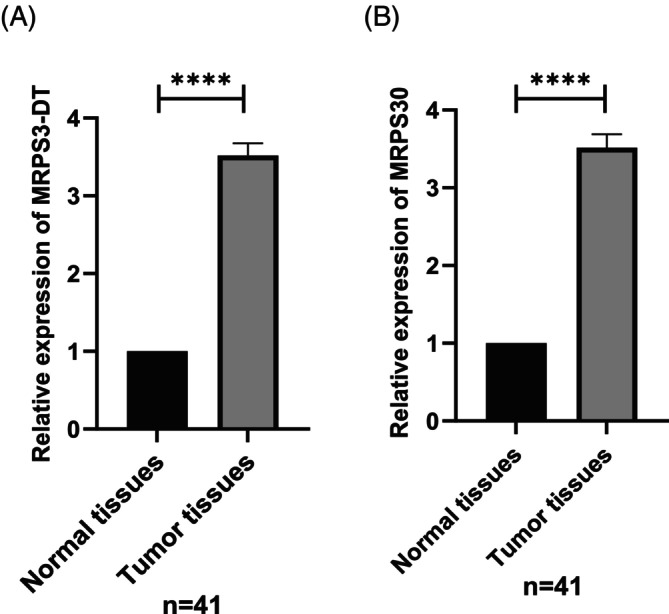
The relative expression of MRPS30‐DT lncRNA and MRPS30 was altered in BC tissues compared with adjacent non‐tumoral tissues. The expression of these parameters was detected by qRT‐PCR technology in 41 BC tissues and paired adjacent tissues. (A) MRPS30‐DT lncRNA was significantly upregulated in breast cancer tissues compared with adjacent normal tissues (*p*‐value <.0001). (B) MRPS30 was also significantly upregulated in breast cancer tissues compared with adjacent normal tissues (*p*‐value <.0001). Results are presented as mean ± SD, based on a two‐tailed *t*‐test, ****p < .0001.

To detect the expression of MRPS30‐DT and MRPS30 in 41 tumors and their associated normal tissues, qRT‐PCR was performed. According to the results of the qRT‐PCR assay, the expression of MRPS30‐DT and MRPS30 were significantly increased in breast cancer tissues compared with adjacent normal tissues (*p*‐value <.0001) (Figure 4).

### 3.11 Association of MRPS30‐DT lncRNA and MRPS30 expression levels with the clinicopathological features

The association of MRPS30‐DT and MRPS30 expression levels with clinicopathological features is shown in Table 5. Table 5 shows that the expression of MRPS30‐DT lncRNA was significantly higher in patients at a higher stage (lll and lV) than in those at a lower stage (l and ll; *p*‐value = .0187; Figure S5a). According to Figure S5b, MRPS30 expression is inversely related to the presence of estrogen receptors and was lower in the ER+ group than in the ER− group (*p*‐value = .0378). As shown in Figure S5c, the expression of MRPS30 was also significantly increased with advancing grade (*p*‐value = .0219). MRPS30 expression level was also negatively associated with patient age (*p*‐value = .018; Figure S5d). However, there were no significant associations between MRPS30‐DT and MRPS30 expression with the other clinicopathologic data.

**TABLE 5:** Correlation of MRPS30‐DT lncRNA and MRPS30 expression with clinicopathological features in BC patients (*n* = 41).

Clinicopathological features	Number of patients (total: 41)	MRPS30‐DT lncRNA		MRPS30	
		Expression level (mean ± SD)	*p*‐value	Expression level (mean ± SD)	*p*‐value
Ages(years)					
<50	23	5.4 ± 0.207	.5533	4.02 ± 0.208	.018^a^
≥50	18	1.23 ± 0.233		2.59 ± 0.235	
Tumor size					
<2 cm	10	5.6 ± 0.36	.1152	19.4 ± 0.333	.8049
2–5 cm	25	2.095 ± 0.199		2.097 ± 0.211	
>5 cm	6	3.12 ± 0.406		1.18 ± o.398	
TNM stage					
I–II	31	2.54 ± 0.178	.0187^a^	3.46 ± 0.198	.374
III–IV	10	3.91 ± 0.314		3.67 ± 0.3152	
Histological grade					
I–II	26	2.4 ± 0.195	.1196	2.17 ± 0.196	0219*
III	15	3.72 ± 0.252		8.05 ± 0.257	
Tumor subtype					
Luminal A	16	1.18 ± 0.292	.7598	0.71 ± 0.302	.4028
Luminal B	14	2.79 ± 0.266		6.95 ± 0.265	
Luminal HER‐2	4	32.55 ± 0.482		23.554 ± 0.472	
TNB	7	5.2 ± 0.365		11.7 ± 0.372	
Lymphatic metastasis					
Present	19	2.25 ± 0.227	.4227	4.75 ± 0.249	.985
Absent	22	3.43 ± 0.212		2.19 ± 0.233	
ER status					
+	34	2.49 ± 0.1716	.7405	2.74 ± 0.1718	.0378^a^
−	7	5.2 ± 0.365		11.7 ± 0.373	
PR status					
+	30	2.1 ± 0.228	.595	3.57 ± 0.182	.2222
−	11	6.31 ± 0.299		3.36 ± 0.298	
Ki67 index					
<20%	16	1.18 ± 0.292	.5119	0.71 ± 0.302	.4096
>20%	25	4.93 ± 0.199		9.77 ± 0.219	

^a^ Significant.

### 3.12 Correlation between expression levels of MRPS30‐DT lncRNA and MRPS30 gene

**FIGURE 5:**
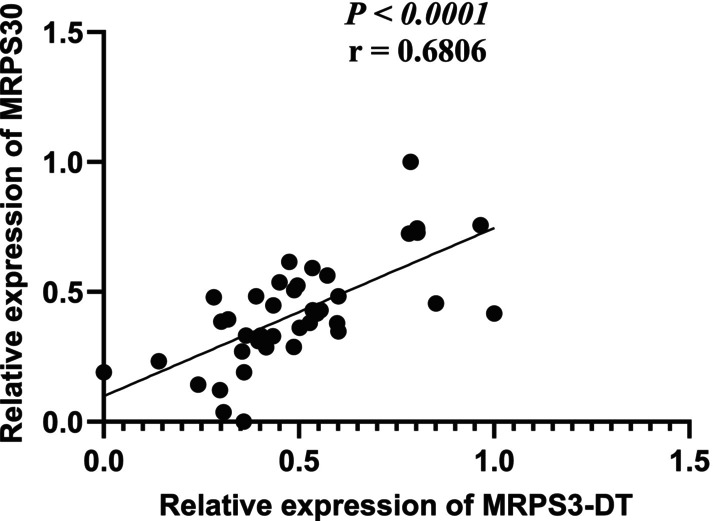
Correlation between relative expression levels of MRPS30‐DT and MRPS30 of tumor tissues based on Pearson correlation test.

A remarkable positive correlation was found between the expression levels of MRPS30‐ DT lncRNA and the MRPS30 gene in tumor tissues (*p*‐value <.0001; *r* = .6806; Figure 5).

### 3.13 The Relative expression level of MRPS30‐DT lncRNA and MRPS30 gene in breast cancer cell lines and HDF cell line

**FIGURE 6:**
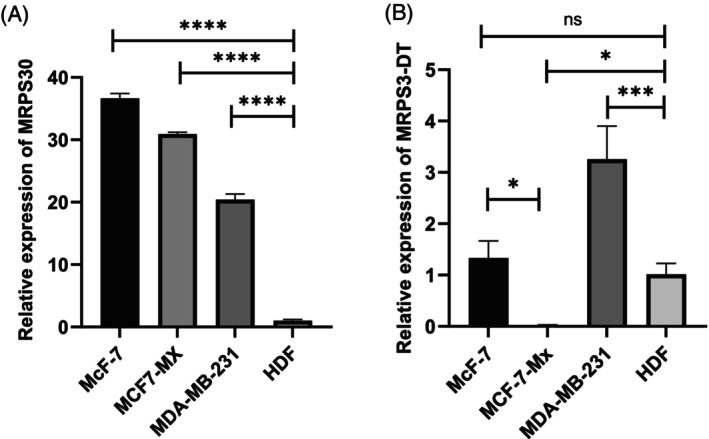
The relative expression level of MRPS30‐DT and MRPS30 in BC cell lines (MCF‐7, MDA‐MB‐231, and MCF‐7/MX) and HDF cell line. (A) There is significant upregulation of MRPS30 in the three breast cancer cell lines MCF‐7, MDA‐MB‐231, and MCF‐7/MX compared to HDF (*p*‐value <.0001). (B) A significant upregulation of MRPS30‐DT in MDA‐MB‐231 compared with HDF (*p*‐value = .0004) and a considerable decrease in MCF‐7/MX compared with HDF and MCF7 (*p*‐value = .049; *p*‐value = .012, respectively) are shown. There was no significant difference in the expression of MRPS30‐DT in MCF‐7 compared with HDF. The results are presented as mean ± SD, based on a one‐way analysis (ANOVA), *p < .05, ***p < .001, ****p < .0001.

In addition, qPCR analysis revealed that the expression level of the lncRNA MRPS30‐DT was significantly upregulated in the cell line MDA‐MB‐231 compared with HDF (*p*‐value <.0004; Figure 6B). While the expression of MRPS30‐DT was considerably reduced in MCF‐7/MX compared with HDF and MCF7 (*p*‐value <.05; Figure 6B). However, when comparing the expression of this lncRNA in MCF‐7 cells compared with HDF, no significant difference in expression was found.

On the other hand, the MRPS30 gene was significantly increased in the three breast cancer cell lines MCF‐7, MDA‐MB‐231, and MCF‐7/MX compared to the normal HDF cell line (*p*‐value <.0001; Figure 6A).

## 4 DISCUSSION

Recent studies have reported that lncRNAs are involved in a wide range of biological and physiological processes and can regulate the main pathways of cancer development at the epigenetic and post‐transcriptional levels.^7^ The data obtained so far have determined that a large number of lncRNAs are involved in the progression of breast cancer and they can be classified into two types: oncogenic and tumor suppressor.^13^, ^39^ However, LncRNAs have recently been identified and most of them have not been investigated yet. Therefore, the development of research in this field is needed and can also contribute to improving diagnosis and even treatment strategies.

In the modern era of clinical medicine, cancer bioinformatics has become an integral component that plays an important role in identifying and evaluating biomarkers. In this study, heatmap, PCA, and DEG analyses were performed using iDEP web software for expression profiles from the TANRIC database. The heatmap and PCA analysis were able to distinguish tumor samples from normal tissue samples, indicating that the data used were appropriate. According to DEG analysis, 18 lncRNAs had increased expression and 46 lncRNAs had decreased expression in tumor tissues compared to normal tissues (FDR <0.01, logFC >1 for upregulated lncRNAs and logFC < −1 for downregulated lncRNAs). MRPS30‐DT was selected as a candidate lncRNA after sharing lncRNAs with increased expression from the TANRIC database and breast cancer‐related lncRNAs from the lncBook database. Since very few studies had been performed on MRPS30‐DT in relation to cancer, especially breast cancer, it was selected as the desired lncRNA for further studies. Using the list of genes co‐expressed with MRPS30‐DT in the lncHUB database, we finally selected the MRPS30 gene as the most related gene.

As limited studies have been conducted on this lncRNA in cancers, analysis of its co‐expressed genes may lead to a better understanding of its function in BC by applying the principle of “guilt by association.” This principle states that the functions, pathways, or expression profiles of a target gene, can be predicted based on co‐expressed genes of it with known functions in relevant biological processes.^40^, ^41^ In the current study, the appyters website was used to perform enrichment analysis of the top 50 genes co‐expressed with MRPS30‐DT lncRNA to investigate its associated signaling pathways, functions, and diseases. According to KEGG pathway analysis, hedgehog signaling and prolactin signaling were the most enriched pathways. The Hedgehog (Hh) signaling pathway has been reported to play a critical role in embryonic development and stem cell differentiation. Much evidence supports the importance of Hh pathway in tumorigenesis and progression of various subtypes of breast cancer, including HR+ and triple‐negative breast cancer TNBC.^42^, ^43^, ^44^ In addition, some studies indicate the role of prolactin and its receptor in the biology of breast cancer.^45^, ^46^ Therefore, MRPS30‐DT might also contribute to the development of breast cancer through these pathways.

GO Enrichment analysis of biological processes revealed that genes co‐expressed with MRPS30‐DT were mainly involved in neuron differentiation and response to interleukin‐4. Evidence suggests that several genes such as Plks may play a role in tumor progression or inhibition by regulating neuronal differentiation.^47^ In addition, IL‐4 is a pleiotropic cytokine that has been associated with proliferation, metastasis, migration, and invasion of breast cancer cells in several studies.^48^ So MRPS30‐DT might be involved in these biological processes. Moreover, there might be a link between MRPS30‐DT and molecular functions such as oncostatin‐M receptor activity and leukemia inhibitory factor receptor activity. Dysregulation of the above functions can lead to the progression of several cancers such as breast cancer.^49^, ^50^ Furthermore, according to CC terms, MRPS30‐DT might be enriched mainly in the protein kinase complex and the HFE‐transferrin receptor complex. The Hfe/Tfr1 protein complex is involved in the regulation of hepcidin transcription, and hepcidin is also involved in the regulation of iron homeostasis. In addition, disturbances in iron metabolism play a role in tumorigenesis, progression, and metastasis of cancer.^51^, ^52^ The protein kinase complex is also crucial for the development of breast cancer. For example, it has been shown that PKCε promotes the survival of breast cancer cells by inhibiting apoptosis and promoting autophagy as well.^53^

Transcriptional enrichment analysis revealed that MRPS30‐DT might be associated the most with ZNF217 and FoxM1, whose roles in breast cancer have been demonstrated in the literature. Studies have demonstrated that ZNF217 acts as an oncogene in breast cancer cell lines by promoting cell proliferation, invasiveness, and chemotherapy resistance.^54^ FoxM1 as an oncogenic transcription factor was associated with poor survival in breast cancer patients and can be used as a potential biomarker for targeted therapies and prognosis assessment in breast cancer patients.^55^, ^56^, ^57^

Disease enrichment analysis of co‐expressed genes revealed that the most enriched diseases were mammary neoplasms, renal cell dysplasia, and breast carcinoma. Thus, this analysis may confirm the role of MRPS30‐DT in breast cancer.

According to the interaction network available on the GeneMANIA website, the MRPS30 gene had the strongest interaction with MLPL37 and MRPL28, respectively. Existing studies have suggested that MRPL37 and MRPL28 may be involved in cell transformation, proliferation, and apoptosis of tumor cells.^58^ According to the protein interactions on the website STRING, the protein relationship between MRPS30 and DAP3 protein was clear, and research shows that DAP3 is required to induce both the extrinsic and intrinsic pathways of apoptosis.^23^, ^58^ According to these network analyses, it is suggested that MRPS30 may play a role in the apoptosis process and breast cancer development through interaction with DAP3, MLPL37, and MRPL28.

According to various reports, lncRNAs and miRNAs are the major regulators of cancer. Moreover, MREs can be shared by lncRNAs and mRNAs, resulting in a competing endogenous RNA network (ceRNA).^59^ The ceRNA is a post‐transcriptional regulatory mechanism for the interaction between RNAs, and its importance in various cancers, such as breast cancer, has recently been demonstrated in numerous studies.^59^, ^60^, ^61^, ^62^, ^63^ Prediction of MRPS30‐DT lncRNA and MRPS30 interaction with miRNAs using the lncBase and TarBase databases led to the identification of five common miRNAs between them, namely hsa‐let‐7b‐5p, hsa‐miR‐1‐3p, hsa‐miR‐16‐5p, hsa‐miR‐34a‐5p, and hsa‐miR‐7‐5p. According to various studies, all of these miRNAs are associated with cancer. For example, let‐7b‐5p inhibits breast cancer cell growth and metastasis by suppressing HK2‐mediated aerobic glycolysis.^64^ Also, miR‐16‐5p has been shown to inhibit breast cancer proliferation through ANLN,^65^ and in another study, its increased expression was associated with inhibition of proliferation, invasion, and induction of apoptosis in breast cancer.^66^ In addition, miR‐34a‐5p and miR‐7‐5p can induce apoptosis and suppress proliferation and migration of breast cancer cells.^67^, ^68^ Also, miR‐1‐3p plays an inhibitory role in cancers such as hepatocellular carcinoma, bladder cancer, and lung adenocarcinoma.^69^, ^70^, ^71^

Therefore, there might be a ceRNA network between MRPS30 lncRNA, MRPS30, and each of these five miRNAs, although further studies are needed.

Following the bioinformatics analysis, we performed in vitro studies with three breast cancer cell lines and one normal HDF cell line and with adjacent tumor and normal tissues from 41 breast cancer patients, which partially confirmed the bioinformatics results. The results of q‐RT PCR in tissues showed that the expression of MRPS30‐DT was significantly increased in tumor tissues compared with adjacent normal tissues. Consistent with our findings, the results of microarray analysis and in situ hybridization analysis of breast cancer and adjacent normal tissues in one study showed an increase in MRPS30‐DT expression levels in tumor tissues compared to normal tissues.^19^ Also, the results of another study in NSCLC showed increased expression of MRPS30‐DT in tumor tissue compared to adjacent normal tissue, as well as in plasma of patients compared to healthy controls and in cancer cells compared to normal cell.^20^ Therefore, in line with our research, mentioned studies can express the role of MRPS30‐DT lncRNA in the development of some cancers. Besides, we found a significant positive association between the expression of MRPS30‐DT and TNM stage, in the way that the expression of MRPS30‐DT was significantly higher in higher stage patients (lll and lV) than in early stage patients (l and ll), while the results of subtype analyses in TANRIC showed no significant association between the expression of MRPS30‐DT and tumor stage. According to a previous study by Balu Wu et al., it was reported that knocking down this lncRNA in MCF‐7 and MDA‐MB‐231 cells increased apoptosis and inhibited proliferation, migration, and invasion of cancer cells.^19^ Thus, it can be concluded that the increased expression of this lncRNA in higher‐stage patients might be due to its role in promoting proliferation, invasion, and migration of breast cancer cells. On the other hand, based on a previous study that showed the role of this lncRNA in activating apoptosis and inhibiting proliferation and migration of NSCLC cells by binding RPS9,^20^ we can speculate that the increased expression of MRPS30‐DT at higher stages might be correlated with the resistance of cells to progressive cancer.

In contrast to our laboratory results which there was no remarkable association between MRPS30‐DT expression and ER and PR status, online TANRIC analyses showed a significant positive association between the MRPS30‐DT expression and the presence of ER and PR. Considering the role of the estrogen receptors ER and PR in various cancers, including breast cancer,^72^, ^73^, ^74^ and the positive association of these receptors with the expression of the lncRNA MRPS30‐DT based on TANRIC online analyses in breast cancer patients, this may suggest a collaboration of MRPS30‐DT and these receptors in the initiation, development, or prevention of breast cancer.

The discrepancy between our in vitro results and the subtype analyses in TANRIC may be due to the different sample volumes; further investigation is required in this regard.

According to q‐RT PCR results in cell lines, MRPS30‐DT was significantly upregulated in the MDA‐MB‐231 compared with the normal HDF cell line. In a previous study, MRP30‐DT was overexpressed in breast tumor tissues,^19^ and its increased expression in breast tumor tissues was also evident in our tissue in vitro results. Therefore, this lncRNA may also be increased in breast cancer cells, as it was increased in MDA‐MB‐231 (triple negative breast cancer cell line) compared with HDF. But, no significant difference was observed in the expression of MRPS30‐DT in MCF‐7 compared to the HDF cell line.

However, the significant reduction of this lncRNA in MCF‐7/MX compared with HDF and MCF7 was interesting. Yang et al., have previously shown that this lncRNA activated apoptosis in NSCLC cells.^20^ Inhibition or reduction of apoptosis is one of the major mechanisms of drug resistance by lncRNAs in cancer cells. Therefore, according to the above study,^20^ reducing the expression of MRPS30‐DT in MCF‐7/MX, a mitoxantrone‐resistant cell line, might play a role in drug resistance by reducing apoptosis.

Overall, MRRPS30‐DT can act as an oncogene or tumor suppressor in breast cancer and might play a role in drug resistance in breast cancer. But, further studies are needed to find out the role and function of this lncRNA in breast cancer development and drug resistance in breast cancer.

In the present study, we have shown significant upregulation of MRPS30 in breast cancer tissues compared to adjacent non‐cancerous tissues. The results of cell analysis also show a significant increase in the expression of this gene in all three cancer cell lines compared to the normal HDF cell line. The importance of the role of MRPS30 gene in breast cancer has been shown in several studies.^27^, ^75^ In another study, Gou et al. showed that MRPS30 plays an important role in breast tumorigenesis by disrupting cell behavior.^24^ Based on the significant increase in the expression of MRPS30 in breast tumor tissues and breast cancer cells in our in vitro studies, in agreement with previous studies, the role of this gene in breast cancer can be confirmed. According to our results, MRPS30 was significantly more expressed in ER‐negative breast cancer patients than in ER‐positive patients. Therefore, we speculate that MRPS30 might contribute to breast cancer development via the steroid hormone receptor pathway. For example, steroid hormone receptors are involved in cell cycle regulatory processes, and cell cycle deregulation causes malignancies of various origins, including breast cancer.^74^ In the current study, we demonstrated that a high expression level of MRPS30 was significantly positively associated with histological grade. It is also known that patients with a higher grade have a more progressive state in the development of breast cancer. In the previous study,^24^ a significant increase in MRPS30 gene expression was observed in MCF‐7, and knockdown of this gene in MCF‐7 also significantly decreased the cell viability of the knockdown cells compared to the control cells. On the one hand, it can be suggested that the higher expression of this gene in the higher grades (lll) than in the lower grades (l and ll) might be due to the effect of this gene in enhancing the viability of cancer cells. On the other hand, as it is clear from the other name of MRPS30 (programmed cell death 9) and according to numerous articles and our bioinformatics results, this gene is one of the pro‐apoptotic genes that plays an important role in the apoptosis process.^23^ Therefore, it can be assumed that the increase in the expression of this gene in the higher grades may be due to combating the progression of breast cancer. In addition, there was a trend toward association between MRPS30 expression and patient age in such a way that expression of this gene tended to be upregulated in <50 years old patients compared with patients ≥50 years of age. The decreased expression of MRPS30 in women aged 50 years and older may be related to menopause and the decline in female hormones.

Because MRPS30 is a pro‐apoptotic gene, its increased expression in breast tumor tissues and cells might indicate cell resistance to these conditions. In this study, as expected from the bioinformatics results, there was a remarkable positive correlation between MRPS30‐DT lncRNA and the MRPS30 gene.

Taken together, the current study sheds light on the dysregulation of MRPS30‐DT lncRNA and MRPS30 in BC. Furthermore, the comprehensive bioinformatics analyses performed in this research could provide a background or general overview of the possible role of these genes in breast cancer. These findings offer new insights into the development of valuable clinical biomarkers for breast cancer. However, due to some shortcomings in our study, further research studies are needed to confirm our findings, determine the exact role of the mentioned genes and investigate their relationship. Firstly, we only had a limited number of clinical samples. Second, further investigations such as cell transfection, cell apoptosis, cell migration and invasion should be performed to clarify the effect of overexpression of MRPS30‐DT lncRNA and MRPS30 in BC. Third, we did not implement in vivo model exploration to validate our in silico and in vitro results.

## 5 CONCLUSION

The results of our in vitro analysis, consistent with the results of our bioinformatics analysis, showed a significant increase in MRPS30‐DT lncRNA expression in tumor tissues compared with normal tissues. In addition, we suggested that MRPS30‐DT may contribute to drug resistance. Therefore, MRRPS30‐DT can exert its role as an oncogene or tumor suppressor in breast cancer development and might play a role in drug resistance in breast cancer. According to various studies, MRPS30 is a pro‐apoptotic gene, so significantly increased expression of this gene in breast tumor tissues and cells might indicate resistance of the cells to these conditions. In agreement with our bioinformatic analysis, we found a significant positive correlation between MRPS30‐DT lncRNA and the MRPS30 gene. Also, based on our in silico studies, MRPS30‐DT lncRNA might compete with miRNA in a ceRNA network for MRPS30 mRNA. Consequently, the current study may provide clues for exploring new therapeutic targets or molecular biomarkers in breast cancer.

## AUTHOR CONTRIBUTIONS

Hajar Yaghoobi supervised the whole project, designed the research strategy, contributed to the analysis (including experiments and statistical analyses), and edited the manuscript. Nooshafarin Shirani participated in material preparation, data collection, experiment performance, data analysis, and manuscript writing. Roohallah Mahdi‐Esferizi and Shahram Tahmasebian contributed to the bioinformatics analysis. Reza Eshraghi Samani helped us to collect the patients' clinical tissues and their information. All authors read and approved the final manuscript.

## FUNDING INFORMATION

This study is financially supported by Shahrekord University of Medical Sciences (grant number: SKUMS‐5231).

## CONFLICT OF INTEREST STATEMENT

The authors have stated explicitly that there are no conflicts of interest in connection with this article.

## ETHICS STATEMENT

The study was conducted according to the guidelines of the Declaration of Consent for publication Shahrekord University of Medical Sciences (SKUMS) (Code: IR.SKUMS.REC.1398.209).

## PATIENT CONSENT FOR PUBLICATION

Informed consent was obtained from all individual participants included in the study.

## Supporting information


**Figure S1.** Heatmap and PCA analysis. **(a)** Heatmap of 12 727 lncRNA expression profiles from the TANRIC database in five breast cancer tissues and five normal tissues. Rows represent lncRNAs and columns represent breast cancer and normal tissues. Relative lncRNA expression is shown using a color scale. Red represents higher expression level and blue represents lower expression level. **(b)** PCA analysis shows different distribution of tumor samples than normal samples. Tumor tissue is indicated by blue circles and normal tissue by red triangles.

**Figure S2.** Different expression patterns of lncRNAs between BC tissues and normal tissues. **(a)** Scatter plot shows the average expression of each lncRNA in tumor and normal tissues in a two‐dimensional image. **(b)** The MA plot provides a quick overview of the distribution of lncRNA expression patterns. Red and blue dots indicate upregulated and downregulated lncRNAs, respectively, while gray dots represent other lncRNAs without significant variation.

**Figure S3.** sharing the data provided from TANRIC and lncBook databases by Venny Diagram.

**Figure S4.** Gene–gene and PPI network of the MRPS30 gene. (**a)** MRPS30 protein association network in SRING database. (**b)** Gene–gene association network of the MRPS30 performed using GeneMANIA.

**Figure S5.** Significant association of MRPS30‐DT lncRNA and MRPS30 expression levels with some clinicopathological features in BC patients. (**a)** MRPS30‐DT lncRNA was significantly upregulated in patients with stage lll&lV than in patients with stage l&ll (*p*‐value = .0187). (**b)** MRPS30 expression in ER+ tumor samples compared to ER‐ tumor samples showed a significant decrease in expression (*p*‐value = .0378). **(c)** MRPS30 expression was significantly lower in grade 1&2 tumor samples compared to grade 3&4 tumor samples (*p*‐value = .0219). (**d)** MRPS30 expression showed upregulation in <50 patients compared to patients ≥50 years (*p*‐value = .018). The results are presented as mean ± SD, *p < .05.

**Table S1.** List of the dysregulated lncRNAs in tumor tissues compared with normal tissues.

**Table S2.** List of top 50 co‐expressed genes with MRPS30‐DT using lncHUB database.

**File S1.** The total list of GO enrichment analysis of biological processes, retrieved from Appyters website (*p*‐value <.05).

**File S2.** The total list of GO enrichment analysis of molecular functions, retrieved from Appyters website (*p*‐value <.05).

**File S3.** The total list of Transcriptional enrichment analysis, retrieved from Appyters website (*p*‐value <.05).

**File S4.** The total list of disease enrichment analysis, retrieved from Appyters website (*p*‐value <.05).

## Data Availability

Data will be made available on reasonable request.

## Endnotes

^*^
Cyclin D1‐interacting long noncoding RNA 1.

^†^
Normal human dermal fibroblast.

^§^
Protein‐protein interaction.
