# Supplementary figures and images for: In silico identification and in vitro evaluation of MRPS30‐DT lncRNA and MRPS30 gene expression in breast cancer
Source: Cancer Rep (Hoboken). 2024 Jun 17;7(6):e2114. doi: 10.1002/cnr2.2114 (PMC11182701; doi:10.1002/cnr2.2114)

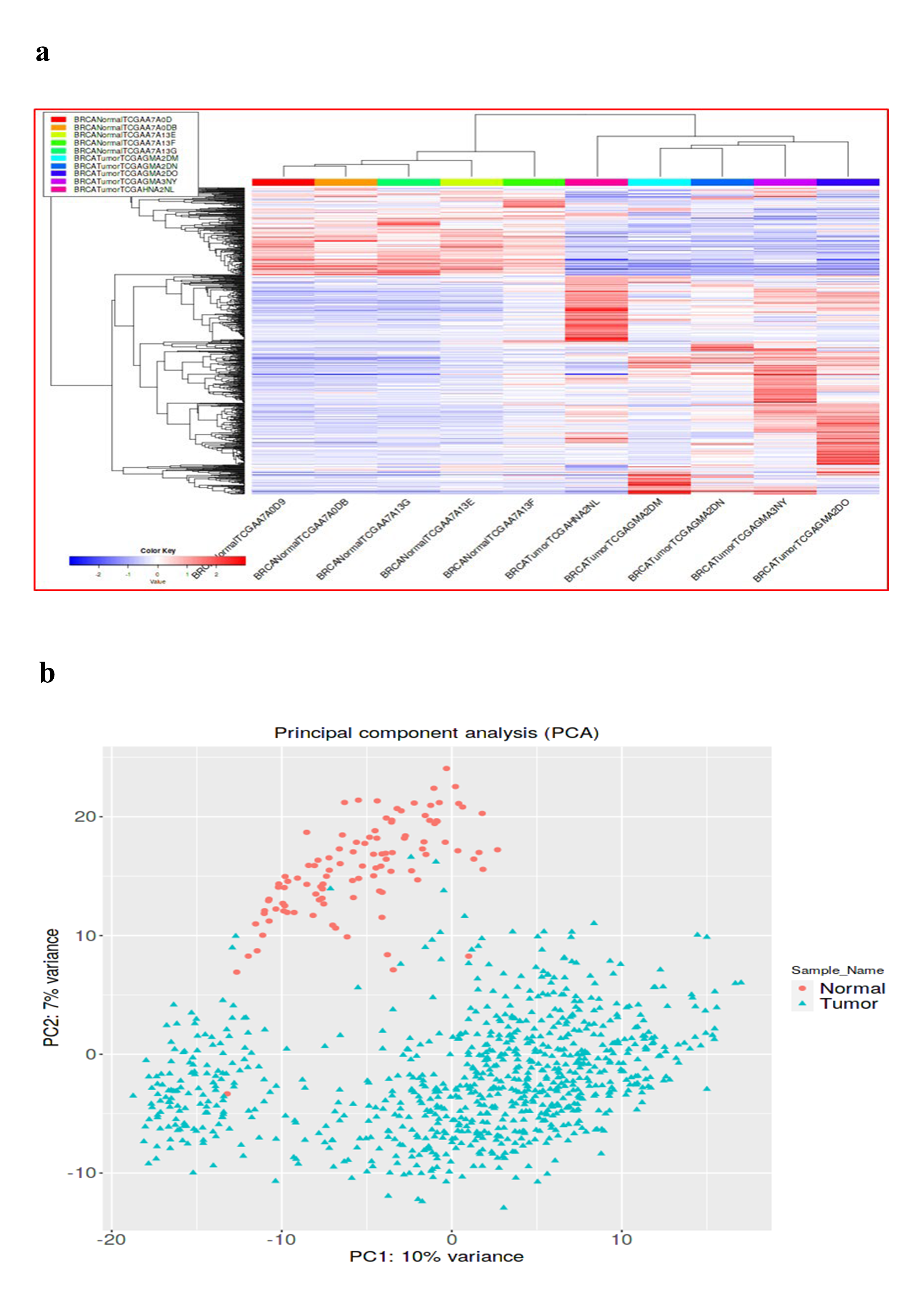

Supplement: Supplementary file 1 — Figure S1. Heatmap and PCA analysis. (a) Heatmap of 12 727 lncRNA expression profiles from the TANRIC database in five breast cancer tissues and five normal tissues. Rows represent lncRNAs and columns represent breast cancer and normal tissues. Relative lncRNA expression is shown using a color scale. Red represents higher expression level and blue represents lower expression level. (b) PCA analysis shows different distribution of tumor samples than normal samples. Tumor tissue is indicated by blue circles and normal tissue by red triangles. [file CNR2-7-e2114-s002.tif]

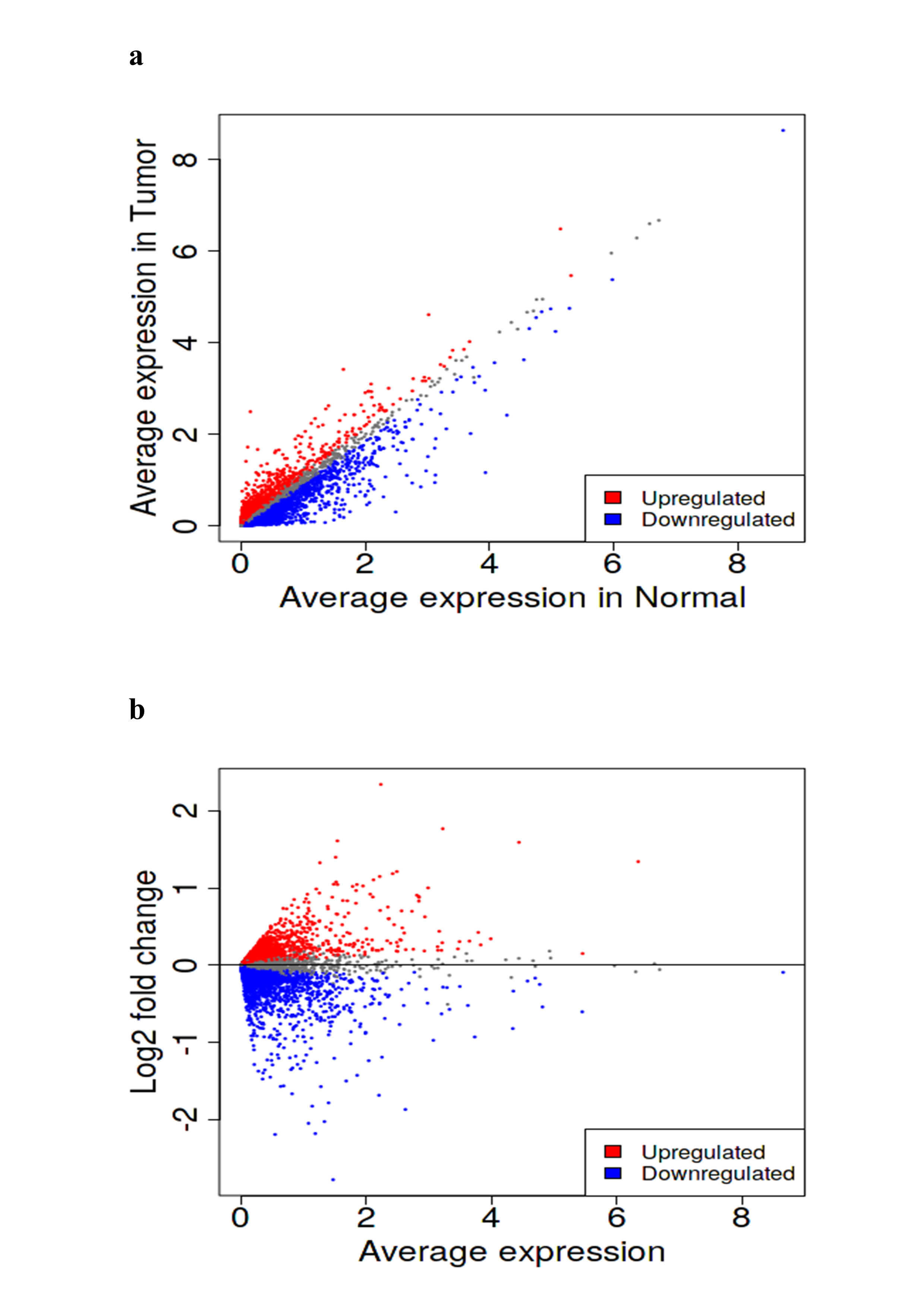

Supplement: Supplementary file 2 — Figure S2. Different expression patterns of lncRNAs between BC tissues and normal tissues. (a) Scatter plot shows the average expression of each lncRNA in tumor and normal tissues in a two‐dimensional image. (b) The MA plot provides a quick overview of the distribution of lncRNA expression patterns. Red and blue dots indicate upregulated and downregulated lncRNAs, respectively, while gray dots represent other lncRNAs without significant variation. [file CNR2-7-e2114-s005.tif]

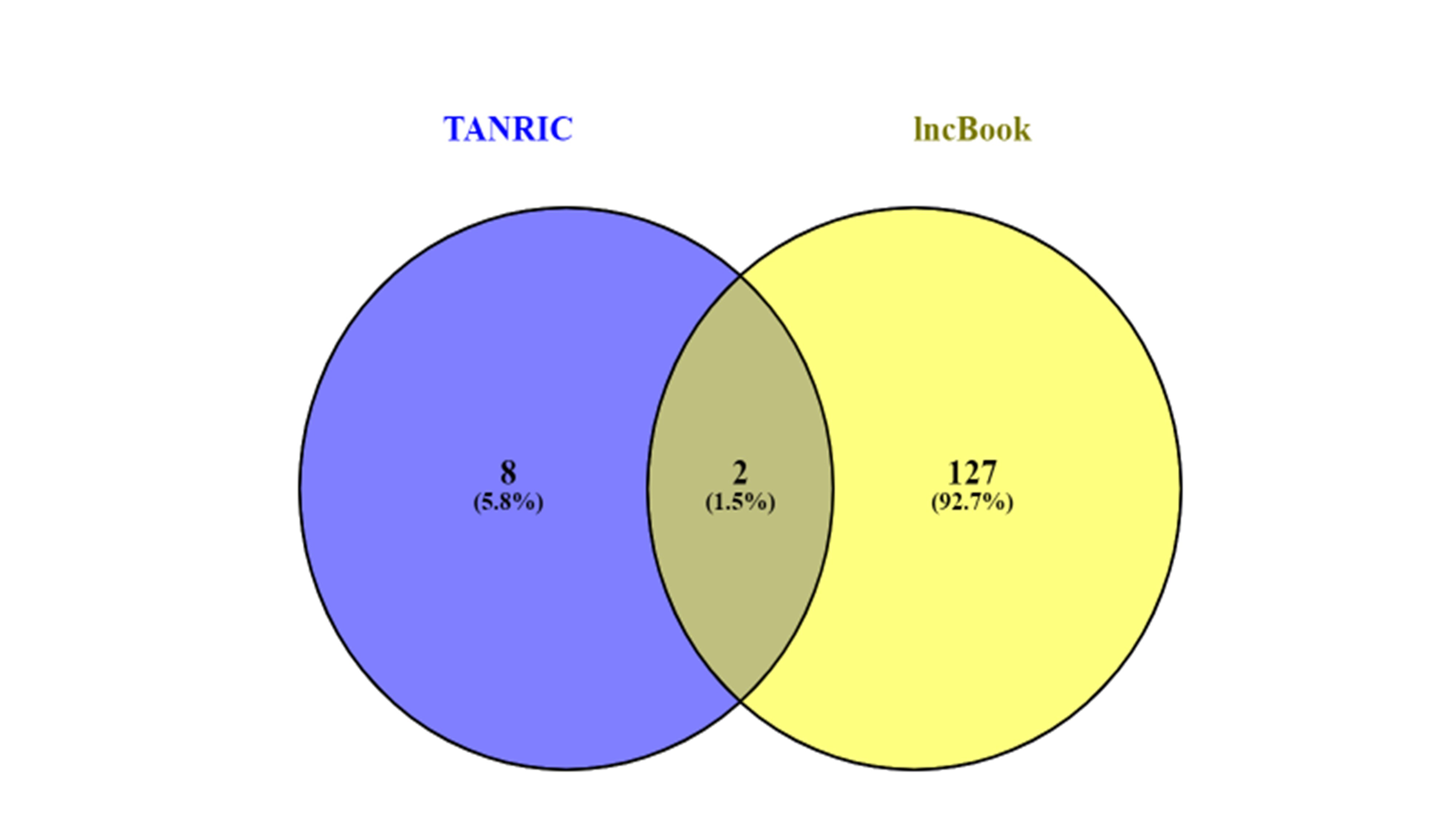

Supplement: Supplementary file 3 — Figure S3. sharing the data provided from TANRIC and lncBook databases by Venny Diagram. [file CNR2-7-e2114-s008.tif]

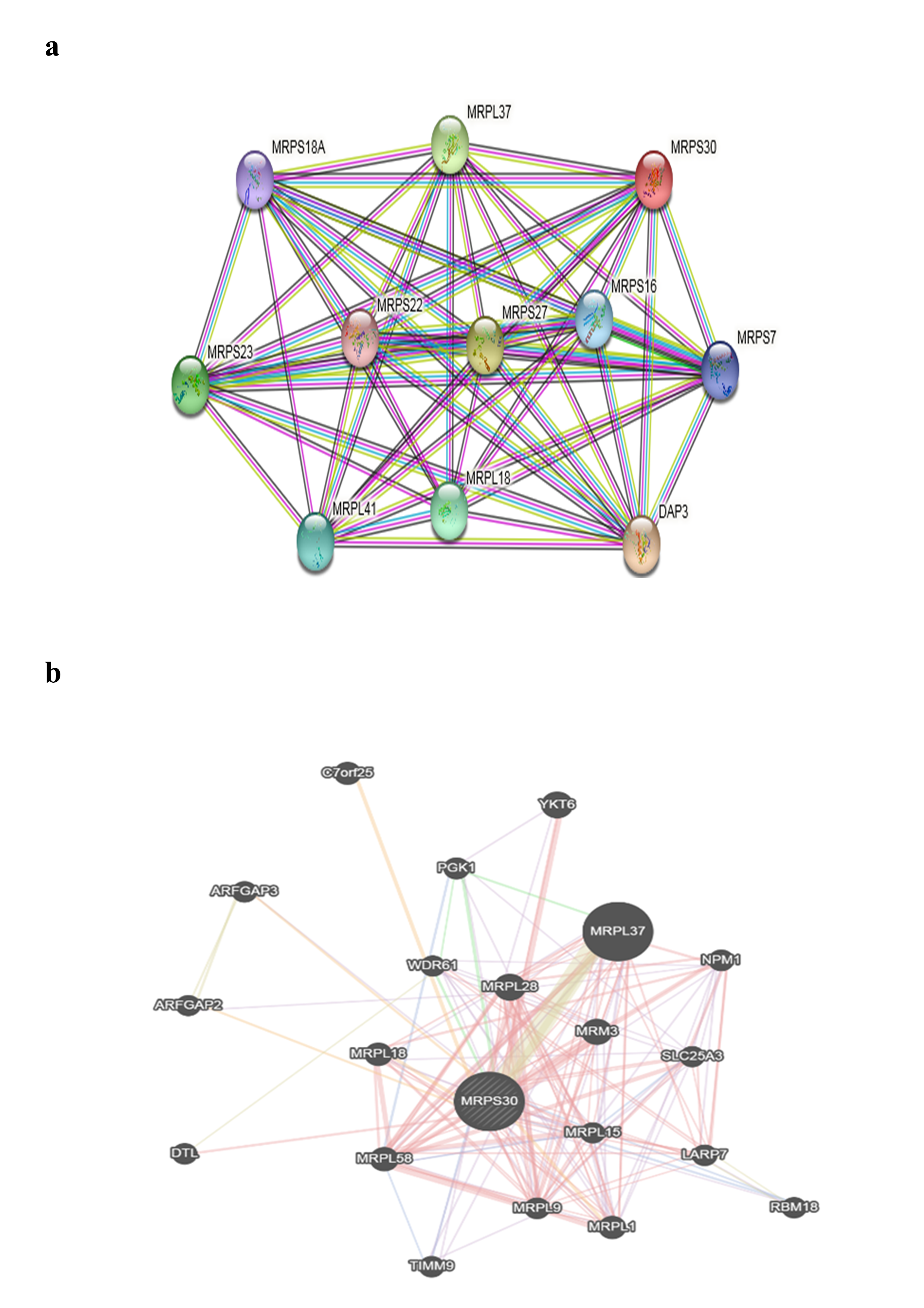

Supplement: Supplementary file 4 — Figure S4. Gene–gene and PPI network of the MRPS30 gene. (a) MRPS30 protein association network in SRING database. (b) Gene–gene association network of the MRPS30 performed using GeneMANIA. [file CNR2-7-e2114-s011.tif]

## Slide 1
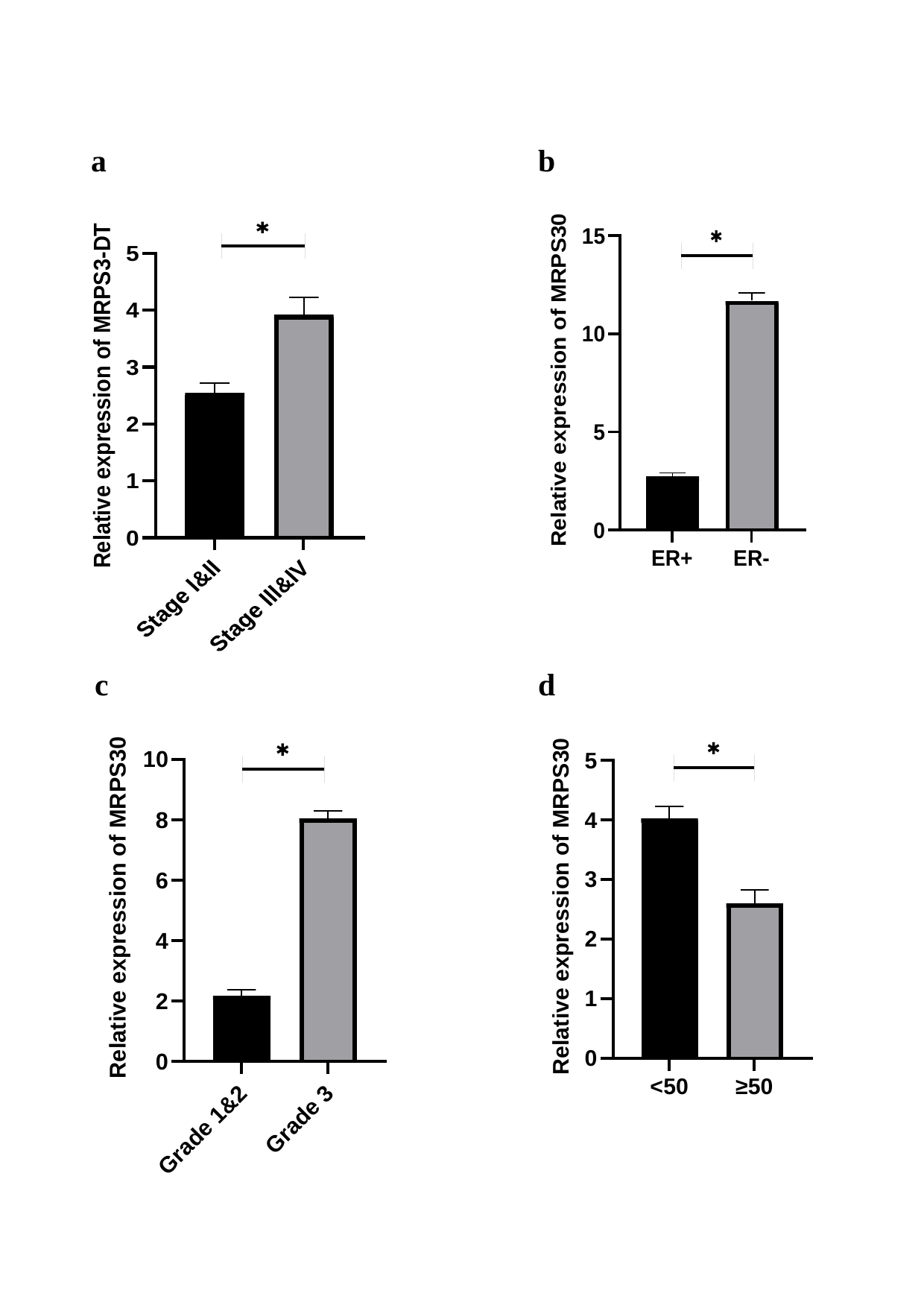

b
a
d
c

Supplement: Supplementary file 5 — Figure S5. Significant association of MRPS30‐DT lncRNA and MRPS30 expression levels with some clinicopathological features in BC patients. (a) MRPS30‐DT lncRNA was significantly upregulated in patients with stage lll&lV than in patients with stage l&ll (p‐value = .0187). (b) MRPS30 expression in ER+ tumor samples compared to ER‐ tumor samples showed a significant decrease in expression (p‐value = .0378). (c) MRPS30 expression was significantly lower in grade 1&2 tumor samples compared to grade 3&4 tumor samples (p‐value = .0219). (d) MRPS30 expression showed upregulation in <50 patients compared to patients ≥50 years (p‐value = .018). The results are presented as mean ± SD, *p < .05. [file CNR2-7-e2114-s009.pptx]
