# Supplementary material for: In silico identification and in vitro evaluation of MRPS30‐DT lncRNA and MRPS30 gene expression in breast cancer
Source: Cancer Rep (Hoboken). 2024 Jun 17;7(6):e2114. doi: 10.1002/cnr2.2114 (PMC11182701; doi:10.1002/cnr2.2114)
Supplement: Supplementary file 6 — Table S1. List of the dysregulated lncRNAs in tumor tissues compared with normal tissues. [file CNR2-7-e2114-s006.docx]

Table S1. List of the dysregulated lncRNAs in tumor tissues compared with normal tissues.

| Upregulated lncRNAs | | | |
| --- | --- | --- | --- |
| Row-names | Symbol | logFC | adj-P-Value |
| ENSG00000230838 | LINC01614 | 2.341821 | 3.75E-79 |
| ENSG00000203499 | IQANK1 | 1.767789 | 2.61E-80 |
| ENSG00000223808 |  | 1.610083 | 1.12E-15 |
| ENSG00000259187 |  | 1.590879 | 4.72E-43 |
| ENSG00000261039 | LINC02544 | 1.398432 | 1.85E-41 |
| ENSG00000258486 | NA | 1.34178 | 1.33E-24 |
| ENSG00000235123 | DSCAM-AS1 | 1.326867 | 4.45E-12 |
| ENSG00000232638 |  | 1.212417 | 9.82E-12 |
| ENSG00000272993 | NA | 1.185802 | 3.73E-36 |
| ENSG00000243350 | NA | 1.148654 | 2.39E-12 |
| ENSG00000233627 | C4A-AS1 | 1.105245 | 6.19E-15 |
| ENSG00000272666 | KLHDC7B-DT | 1.077677 | 1.97E-18 |
| ENSG00000273272 |  | 1.048412 | 4.86E-18 |
| ENSG00000197308 | GATA3-AS1 | 1.044586 | 7.47E-13 |
| ENSG00000223573 | TINCR | 1.044337 | 1.73E-16 |
| ENSG00000261716 | H2BC20P | 1.026047 | 7.59E-34 |
| ENSG00000251141 | MRPS30-DT | 1.020542 | 7.34E-07 |
| ENSG00000268913 | NA | 1.001295 | 1.07E-21 |
| Downregulated lncRNA | | | |
| Row-names | Symbol | logFC | adj-P-Value |
| ENSG00000228223 | HCG11 | -1.017371351 | 8.51E-52 |
| ENSG00000235387 | SPAAR | -1.020539284 | 7.69E-58 |
| ENSG00000228162 |  | -1.043000128 | 1.41E-112 |
| ENSG00000272639 |  | -1.05048961 | 5.90E-48 |
| ENSG00000256916 |  | -1.069704648 | 3.83E-25 |
| ENSG00000264868 | NA | -1.080167322 | 1.25E-26 |
| ENSG00000250538 |  | -1.096920898 | 1.21E-102 |
| ENSG00000243836 | WDR86-AS1 | -1.151180613 | 1.73E-114 |
| ENSG00000178947 | SMIM10L2A | -1.161204888 | 3.38E-102 |
| ENSG00000267194 | NA | -1.162956703 | 3.51E-80 |
| ENSG00000233429 | HOTAIRM1 | -1.179118648 | 8.08E-86 |
| ENSG00000271738 | NA | -1.191983317 | 5.99E-56 |
| ENSG00000214548 | MEG3 | -1.199354187 | 6.28E-108 |
| ENSG00000268164 | NA | -1.204434904 | 2.00E-91 |
| ENSG00000260025 | CRIM1-DT | -1.20586992 | 2.24E-61 |
| ENSG00000245812 | LINC02202 | -1.227351365 | 9.13E-187 |
| ENSG00000262179 | MYMX | -1.233789324 | 8.47E-97 |
| ENSG00000186594 | MIR22HG | -1.237175737 | 3.42E-84 |
| ENSG00000270547 | LINC01235 | -1.239824501 | 1.50E-40 |
| ENSG00000255248 | MIR100HG | -1.261015808 | 3.81E-98 |
| ENSG00000272143 | FGF14-AS2 | -1.285565793 | 8.60E-98 |
| ENSG00000236333 | TRHDE-AS1 | -1.286805825 | 3.49E-154 |
| ENSG00000257877 | NA | -1.291455467 | 1.15E-136 |
| ENSG00000267532 | MIR497HG | -1.332087846 | 2.49E-144 |
| ENSG00000234456 | MAGI2-AS3 | -1.339265343 | 4.57E-157 |
| ENSG00000272327 | NA | -1.352076922 | 4.12E-66 |
| ENSG00000258545 | RHOXF1-AS1 | -1.356631857 | 9.13E-112 |
| ENSG00000267653 |  | -1.372853753 | 2.03E-106 |
| ENSG00000229108 | LINC02587 | -1.395635686 | 3.40E-161 |
| ENSG00000230148 | HOXB-AS1 | -1.426672474 | 6.87E-41 |
| ENSG00000228971 | LINC02607 | -1.450759321 | 3.20E-106 |
| ENSG00000249669 | CARMN | -1.474915257 | 1.72E-230 |
| ENSG00000230937 | MIR205HG | -1.500142384 | 1.65E-26 |
| ENSG00000180139 | ACTA2-AS1 | -1.563395501 | 2.32E-116 |
| ENSG00000231367 | LINC02613 | -1.570416598 | 3.26E-81 |
| ENSG00000258663 |  | -1.573129653 | 3.39E-94 |
| ENSG00000255471 |  | -1.665847283 | 5.51E-149 |
| ENSG00000267519 | MIR23AHG | -1.684869673 | 1.98E-79 |
| ENSG00000267047 |  | -1.783333068 | 1.84E-107 |
| ENSG00000238018 |  | -1.826091508 | 2.21E-140 |
| ENSG00000272761 | NA | -1.868752854 | 7.90E-46 |
| ENSG00000229645 | NA | -2.025990744 | 1.31E-131 |
| ENSG00000228639 | ROCR | -2.048436659 | 1.68E-42 |
| ENSG00000254148 | NA | -2.181617437 | 1.71E-43 |
| ENSG00000227591 | HSD11B1-AS1 | -2.192798277 | 1.12E-166 |
| ENSG00000269936 | NA | -2.778459057 | 3.76E-167 |
