# Supplementary material for: In silico identification and in vitro evaluation of MRPS30‐DT lncRNA and MRPS30 gene expression in breast cancer
Source: Cancer Rep (Hoboken). 2024 Jun 17;7(6):e2114. doi: 10.1002/cnr2.2114 (PMC11182701; doi:10.1002/cnr2.2114)
Supplement: Supplementary file 7 — Table S2. List of top 50 co‐expressed genes with MRPS30‐DT using lncHUB database. [file CNR2-7-e2114-s004.docx]

Table S2. List of top 50 co-expressed genes with MRPS30-DT using lncHUB database.

| score | Gene symbol | Rank | score | Gene symbol | Rank |
| --- | --- | --- | --- | --- | --- |
| 0.389 | FSIP1 | 26 | 0.916 | MRPS30 | 1 |
| 0.381 | IRX5 | 27 | 0.526 | ESR1 | 2 |
| 0.380 | PKIB | 28 | 0.506 | XBP1 | 3 |
| 0.377 | RAB30 | 29 | 0.505 | GATA3 | 4 |
| 0.376 | PRLR | 30 | 0.505 | BMPR1B | 5 |
| 0.374 | KCTD3 | 31 | 0.496 | TBC1D9 | 6 |
| 0.372 | CT62 | 32 | 0.488 | RABEP1 | 7 |
| 0.370 | KIAA0040 | 33 | 0.465 | TRPS1 | 8 |
| 0.365 | TMEM26 | 34 | 0.465 | KCNE4 | 9 |
| 0.365 | SPOPL | 35 | 0.461 | ELP2 | 10 |
| 0.362 | RGS22 | 36 | 0.446 | TPRG1 | 11 |
| 0.356 | FOXA1 | 37 | 0.430 | SLC39A6 | 12 |
| 0.355 | MAGED2 | 38 | 0.428 | ELOVL5 | 13 |
| 0.354 | GFRA1 | 39 | 0.423 | NBPF6 | 14 |
| 0.354 | CLSTN2 | 40 | 0.421 | LMX1B | 15 |
| 0.351 | ZNF396 | 41 | 0.420 | ZNF552 | 16 |
| 0.350 | UGCG | 42 | 0.420 | SCUBE2 | 17 |
| 0.348 | ZNF92 | 43 | 0.418 | NBPF4 | 18 |
| 0.348 | NEK10 | 44 | 0.414 | AFF3 | 19 |
| 0.348 | AGR3 | 45 | 0.412 | FGF10 | 20 |
| 0.347 | BTRC | 46 | 0.407 | ARMT1 | 21 |
| 0.346 | SUSD3 | 47 | 0.405 | IL6ST | 22 |
| 0.345 | AGTR1 | 48 | 0.404 | STC2 | 23 |
| 0.344 | PTPRT | 49 | 0.401 | FAM214A | 24 |
| 0.344 | CCDC74A | 50 | 0.400 | COX6C | 25 |
